# E2 Glycoprotein of BVDV 1u Circulating in Qinghai Yaks: A Candidate for Serological Surveillance and Subunit‐Vaccine Development With Neutralizing‐Antibody Evaluation in Yaks

**DOI:** 10.1155/tbed/3180012

**Published:** 2026-10-21

**Authors:** Jie Li, Ru Meng, Zhi Li, Yuan Han, Jinshu Sui, Shuai Wang, Shandian Gao, Yong Fu

**Affiliations:** ^1^ Academy of Animal Science and Veterinary Medicine Qinghai University Xining 810016 China qhu.edu.cn; ^2^ Xining Animal Disease Prevention and Control Center Xining 810003 China; ^3^ State Key Laboratory of Animal Disease Prevention and Control Lanzhou Veterinary Research Institute Chinese Academy of Agricultural Sciences Lanzhou 730046 China caas.cn

**Keywords:** bovine viral diarrhea virus (BVDV), BVDV 1u, E2 protein, immunogenicity, indirect ELISA, neutralizing-antibody evaluation, serosurveillance, vaccine antigen, yak

## Abstract

Bovine viral diarrhea (BVD) virus (BVDV) is a globally distributed *Pestivirus* that severely affects the cattle industry, and *Pestivirus bovis* (formerly BVDV‐1), subgenotype 1u is the predominant subtype circulating in yak populations of Qinghai, China. This study aimed to express the N‐terminal fragment (amino acids 1–200) of the E2 glycoprotein of yak‐origin BVDV 1u and identify its antigenic and immunogenic properties for potential application in serological surveillance and subunit‐vaccine development. Using the purified truncated E2 antigen, an indirect ELISA was established for detection of BVDV‐specific antibodies from yak sera. Immunogenicity for the recombinant protein was evaluated in BALB/c mice using Freund’s adjuvant and Montanide ISA 201 VG by determining CD4^+^/CD8^+^ T‐cell activation and the secretion of IFN‐γ, TNF‐α, and IL‐6. Immunization was further performed in BVDV‐naïve yak calves to assess BVDV 1u‐specific neutralizing antibody responses. The established indirect ELISA achieved a diagnostic sensitivity of 99.0 % and specificity of 97.4 %, without detectable cross‐reactivity against other common bovine pathogens; screening of 377 field yak serum samples yielded a BVDV seroprevalence of 21.48 %. Both adjuvant formulations elicited high antibody levels in mice, yet Montanide ISA 201 VG induced stronger T‐cell activation and proinflammatory cytokine production. In immunized yak calves, the E2‐ISA 201 vaccine antigen elicited BVDV 1u‐specific neutralizing antibody titers that showed trends similar to those induced by commercial inactivated vaccines. Our findings demonstrate that the truncated BVDV 1u E2 serves as a feasible antigen for serological surveillance and a candidate subunit‐vaccine development for BVDV. This study provides reliable experimental evidence for regional BVDV prevention and control in yak‐rearing areas of China.

## 1. Introduction

Bovine viral diarrhea (BVD) is a devastating infectious disease of ruminants caused by BVD virus (BVDV), which belongs to the genus *Pestivirus* within the family Flaviviridae. The disease is listed as a notifiable disease by the World Organization for Animal Health (WOAH) and classified as a Class III animal disease by the Ministry of Agriculture and Rural Affairs of China. BVDV has a broad host range beyond domestic cattle, capable of infecting numerous domestic and wild ruminants, including sheep, goats, white‐tailed deer, mule deer, elands, chevrotains, and alpacas [1–5]. BVDV causes severe damage to breeding cattle. In addition to inducing early abortion, stillbirth, and fetal malformation, BVDV can cross the placental barrier and infect fetuses, leading to the production of persistently infected (PI) calves and causing substantial, long‐term economic losses to the global cattle industry [6, 7].

The BVDV genome is ~12.3 kb in length and consists of a single‐stranded positive‐sense RNA, containing a 5^′^‐untranslated region (5^′^‐UTR), one open reading frame (ORF), and a 3^′^‐UTR [8]. The ORF encodes a large polyprotein that is posttranslationally processed into four structural proteins (C/E^rns^/E0/E2) and eight nonstructural proteins (N^pro^, p7, NS2, NS3, NS4A, NS4B, NS5A, and NS5B) [9]. Based on its cytopathic (CP) characteristics in infected cells, BVDV is classified into two biotypes: noncytopathic (NCP) and CP [10]. BVDV has been divided into BVDV‐1 and BVDV‐2, corresponding to the species *Pestivirus bovis* and *Pestivirus tauri*, respectively, and HoBi‐like *Pestivirus* (historically termed BVDV‐3) represents a distinct *Pestivirus* species, *Pestivirus brazilense*, which has recently been detected in South America, Europe, and Asia [11, 12]. Phylogenetic analysis of genomic regions, including the 5^′^‐UTR, Npro, and E2 facilitates further classification of BVDV into multiple subgenotypes [13].

China possesses the world’s predominant yak resource, with a population exceeding 16 million, accounting for more than 90% of the global total. In recent years, with the expansion of yak husbandry and intensified epidemiological surveillance, increasing studies have focused on the epidemiology and pathogenic mechanisms of BVDV infection in yaks [14]. BVDV genotypes exhibit a marked variation in their geographic distribution worldwide. BVDV‐1a, 1b, and 2a are prevalent in the United States, BVDV‐1b is endemic in Europe, while BVDV‐1 c is predominantly restricted to Oceania; BVDV‐2 comprises multiple subgenotypes, including 2a, 2 b, 2 c, and 2d, with BVDV‐2a serving as the globally dominant strain [15]. A national epidemiological analysis revealed that the overall prevalence of BVDV in China is 9.8%, with a significantly higher infection rate in yaks than that in beef and dairy cattle [16]. A wide range of BVDV subgenotypes circulate in China, including BVDV‐1a–1d, 1 m–1q, 1u–1 w, and BVDV‐2a–2 b. Among them, BVDV‐1 m is currently the most prevalent subgenotype nationwide. In addition to the prevalent BVDV‐1 subgenotypes, BVDV‐2 is gradually spreading in Jiangsu, Shandong, Henan, Shaanxi, and Xinjiang [17]. Furthermore, the BVDV‐3 strain has been isolated from cattle, goats, and sheep in Henan, Shandong, and Xinjiang, indicating the emergence and cross‐species transmission of this genotype in China [18]. Of note, Qinghai is the largest yak husbandry region across China, and our recent surveillance data confirmed that BVDV 1u was the predominant subtype circulating in local yaks [19].

E2 is a surface glycoprotein embedded in the BVDV envelope. This protein principally facilitates viral attachment and invasion of host cells and can trigger specific neutralizing antibody responses, conferring strong immunogenic properties [20]. Owing to the high conservation of its antigenic domains, cross‐reactivity during antigen–antibody reactions is significantly minimized, thereby ensuring high accuracy in viral detection [21]. Previous studies have mainly focused on the immunological evaluation of full‐length BVDV E2 protein, while few studies have investigated the immune responses of truncated BVDV 1u E2 in yak calves. Given the above studies, bioinformatics analysis on the E2 protein of BVDV 1u, the dominant subtype among yaks in Qinghai Province, was conducted, and a recombinant prokaryotic vector to express this truncated protein in *Escherichia coli* was obtained. Moreover, we established an indirect ELISA method based on the E2 antigen for BVDV serological surveillance and evaluated its immunogenicity in mice and yaks. Our findings lay a solid theoretical foundation for the development of BVDV diagnostic reagents and subunit vaccine.

## 2. Materials and Methods

### 2.1. Bioinformatics Analysis of BVDV 1u E2 Protein

The E2 gene fragment was successfully amplified from yak‐derived BVDV 1u using E2‐specific primers [22] and the PrimeScript One Step RT‐PCR Kit Ver.2 (Dye Plus) (TaKaRa, Dalian, China, #RR057A). The amino acid composition and physicochemical properties of E2 were analyzed using the online tool ProtParam (https://web.expasy.org/protparam/) [23]. The transmembrane regions were predicted with TMHMM‐2.0 (https://services.healthtech.dtu.dk/services/TMHMM-2.0/) [24]. SignalP‐5.0 (https://services.healthtech.dtu.dk/services/SignalP-5.0/) [25] was employed to predict the presence of a signal peptide. The secondary structure was predicted using SOPMA on the ExPASy website (https://npsa.lyon.inserm.fr/cgi-bin/npsa_automat.pl?page=/NPSA/npsa_sopma.html) [26], and the tertiary structure of BVDV 1u E2 protein was predicted using SWISS‐MODEL (https://swissmodel.expasy.org/).

### 2.2. Design and Synthesis of the Recombinant Expression Vector for E2

Based on bioinformatic predictions, the transmembrane domain of the E2 protein was truncated and excluded from the recombinant construct. The polypeptide covering amino acid residues 1–200 of the E2 protein was selected as the target sequence for prokaryotic expression. Two restriction enzymes, FastDigest *Bam*HI and FastDigest *Hin*dIII (Thermo Scientific, USA, #FD0054 and #FD0505), were selected for recombinant prokaryotic vector construction. The artificially synthesized gene was inserted into the prokaryotic expression vector, pET‐28a. The recombinant plasmid was confirmed by restriction double digestion and sequencing and designated as pET‐28a‐E2.

### 2.3. Expression and Solubility Analysis of the Recombinant E2 Protein

The pET‐28a‐E2 plasmid was transformed into a Transetta (DE3) chemically competent cell (TransGen Biotech, Beijing, China, #CD801−02). A single colony was picked up and inoculated into a LB liquid medium supplemented with 0.1 g/L kanamycin. The culture was incubated at 37°C with shaking until the OD_600_ value reached 0.6–0.8. To induce targeted protein expression, IPTG was added at a final concentration of 1 mmol/L. The cultures were incubated on a shaker for 6 h, and an untreated control was prepared concurrently. Bacterial cells were harvested and resuspended in PBS, followed by ultrasonic disruption. After high‐speed centrifugation, the supernatant and pellet were collected separately and analyzed by SDS‐PAGE to identify the expression pattern of the target protein. Subsequently, western blot was performed to confirm the target protein expression. The primary antibody was mouse anti‐His tag monoclonal antibody (Abcam, USA, #ab18184), and the secondary antibody was horseradish peroxidase (HRP)‐conjugated goat anti‐mouse IgG (Abcam, USA, #ab6789).

### 2.4. Purification and Reactivity Identification of the Target Protein

Following ultrasonic lysis, the bacterial pellets were sequentially washed with PBS and urea‐free IB wash buffer. The mixture was centrifuged at 9000 rpm for 10 min, and the supernatant was discarded. The pellet was rewashed with IB wash buffer containing 4 mol/L urea and centrifuged, and the supernatant was discarded. Elution buffer with 6 mol/L urea and 20 mM imidazole was then added. The suspension was sonicated and incubated overnight at 4°C on a shaker to allow for complete solubilization. The mixture was centrifuged at 8000 rpm for 30 min, and the supernatant was harvested and filtered through a 0.22 μm filter. The filtrate was loaded onto an affinity column and incubated with shaking at 4°C for 1.5 h to facilitate protein binding, after which the flow‐through fraction was collected. Impurity proteins were removed by stepwise elution using 6 mol/L urea buffer with gradient‐increased imidazole concentrations, and the target protein was finally eluted with an elution buffer containing 6 mol/L urea and 500 mM imidazole. Then, western blot was used to assess the antigenic activity of the recombinant protein. BVDV‐positive serum collected from yaks (dilution 1:100) was used for primary antibody, and HRP‐labeled goat anti‐bovine IgG (dilution 1:10,000) (Abcam, USA, #ab102154) was used as the secondary antibody.

### 2.5. Establishment and Evaluation of an Indirect ELISA for Detecting Antibodies Against BVDV

The chessboard titration assay was used to optimize different reaction conditions for the indirect ELISA, such as antigen coating concentration, serum dilution ratio, blocking reagent, working concentration of HRP‐labeled secondary antibody, and incubation parameters. Standard positive and negative serum samples were included as parallel controls throughout the optimization process to validate experimental reproducibility. To determine the cutoff value of the established indirect ELISA, a total of 463 yak serum samples with confirmed BVDV antibody status (positive or negative) were determined by using the ID Screen BVD p80 Antibody Competition Kit (IDvet Innovative Diagnostics, France, #BVDC‐5 P). The OD_450 nm_ values were measured, and the percentage positivity (PP) was calculated using the following formula:

PP=meanOD450 nm of test sample−meanOD450 nm of negative controlmeanOD450 nm of positive control−meanOD450 nm of negative control×100%.

A receiver operating characteristic (ROC) curve was plotted to identify the optimal cutoff value. Serially diluted BVDV‐positive sera (*n* = 3) were subjected to the established indirect ELISA to determine the analytical sensitivity. Sera positive for other bovine pathogens, including bovine *Rotavirus*, infectious bovine rhinotracheitis virus, *Pasteurella multocida*, *Mycoplasma bovis*, and foot‐and‐mouth disease virus, were used for specificity verification. Four serum samples were tested in triplicate to assess intra‐ and interassay reproducibility, which was quantified by calculating the coefficient of variation. Finally, in addition to the 463 yak sera samples used for cutoff value determination, another 377 yak serum samples collected from different regions of Qinghai Province were applied for the clinical evaluation of the established indirect ELISA.

### 2.6. Immunization of Mice With the Recombinant Protein and Determination of Serum Antibody Levels

Twelve female BALB/c mice aged 6–8 weeks were purchased from the Laboratory Animal Center of the Lanzhou Veterinary Research Institute, Chinese Academy of Agricultural Sciences. The mice were randomly divided into three experimental groups. For the first immunization, 20 μg recombinant E2 protein was emulsified 1:1 with Freund’s complete adjuvant (Sigma–Aldrich, USA, #F5881) or Montanide ISA 201 VG (SEPPIC, France). The preparations were delivered via subcutaneous injection on the back to establish the E2‐FA (E2 protein + Freund’s adjuvant) and E2‐201 (E2 protein+ Montanide ISA 201 VG) groups. Mice in the control group were immunized with PBS emulsified at a 1:1 ratio with Freund’s adjuvant (Sigma–Aldrich, USA, #F5881). Two booster immunizations were carried out every 14 days. The E2 protein was formulated with Freund’s incomplete adjuvant (Sigma–Aldrich, USA, #F5506) or Montanide ISA 201 VG (SEPPIC, France) at a 1:1 ratio for the E2‐FA and E2‐201 groups, respectively, using the same dosage as the primary immunization. Fourteen days after the third immunization, the blood samples from different groups were collected, and serum was separated for antibody level analysis derived from mice against BVDV 1u E2 protein.

An indirect ELISA assay was performed to detect and quantify antibody sensitivity against the E2 protein. The ELISA microplate was coated with 23.60 μg/mL E2 protein (100 μL/well) and incubated at 37°C for 2 h. After three PBST washes, blocking was carried out for 30 min at 37°C. Polyclonal antibodies were serially diluted from 1:2000 and loaded in duplicate. The plate was incubated with HRP‐labeled goat anti‐mouse IgG (1:20,000) (Abcam, USA, #ab6789) after washing. TMB substrate (Elabscience, Wuhan, China, #E‐IR‐R201) was added for the chromogenic reaction in the dark at room temperature for 10 min, and the reaction was quenched with stop solution (Elabscience, Wuhan, China, #E‐ELIR‐006). The absorbance at OD_450 nm_ was detected using a microplate reader.

### 2.7. Specificity Detection of Polyclonal Antibodies

To verify specific binding of the E2 protein to its corresponding polyclonal antibodies, the purified recombinant E2 protein was separated by SDS‐PAGE and transferred to a PVDF membrane for western blotting analysis. The E2 polyclonal antibody (dilution 1:500) and HRP‐conjugated goat anti‐mouse IgG (Abcam, USA, #ab6789) (dilution 1:10,000) served as the primary antibody and the secondary antibody, respectively.

To further evaluate the binding reactivity of the obtained polyclonal antibody to BVDV 1u (BVDV‐2024‐yak‐1 strain) [27], MDBK cells were seeded into confocal dishes and incubated overnight at 37°C in a 5% CO_2_ incubator. The cells were incubated with BVDV 1u for 12 h before the removal of the supernatant. Subsequently, the cells were fixed with 4% paraformaldehyde for 30 min at room temperature, washed three times using PBS, and blocked with 3% BSA‐PBS for 1 h at room temperature. Following three more washes in PBS, the cells were treated with E2 polyclonal antibody derived from mice at a dilution of 1:200 for 1 h at 37°C. Mouse negative serum (dilution 1:200) and PBS were used as the negative control and mock group, respectively. Subsequently, the cells were washed three times with PBS and incubated with Alexa Fluor 488‐conjugated goat anti‐mouse IgG H&L (1:1000) (Thermo Scientific, USA, #A28175) for 30 min at room temperature in darkness. After five PBS washes, DAPI solution (Thermo Scientific, USA, #62248) was added and incubated for 10 min in the dark. After a final rinse, cells were visualized via laser scanning confocal microscopy.

### 2.8. Cytokine Detection

Fourteen days after the third immunization with the recombinant E2 protein, serum specimens were isolated from immunized mice. The serum contents of IFN‐γ, TNF‐α, and IL‐6 were measured via the LEGEND MAX Mouse IFN‐γ ELISA Kit (BioLegend, USA, #430807), LEGEND MAX Mouse TNF‐α ELISA Kit (BioLegend, USA, #430907), and LEGEND MAX Mouse IL‐6 ELISA Kit (BioLegend, USA, #431307), respectively. GraphPad Prism 10.1.2 software was used for intergroup statistical analysis.

### 2.9. Detection of T Cell Activation in Mouse Splenic Tissues

Fourteen days after receiving the third E2 protein immunization, the mice were euthanized. Spleens were harvested aseptically and placed in sterile Petri dishes. After adding 5 mL of RPMI‐1640 medium, the spleens were gently triturated using the plunger of a 1 mL syringe. The cell suspension was filtered through a 70 μm nylon mesh into a sterile 50 mL centrifuge tube and then centrifuged at 500 × *g* for 5 min at 4°C. Most of the supernatant was aspirated, leaving about 2 mL of liquid, and the cell pellet was transferred to a 15 mL centrifuge tube. The pellet was resuspended in 4 mL of red blood cell lysis buffer and incubated at room temperature for 5 min. Lysis was terminated by adding staining buffer, and the cells were centrifuged again at 500 × *g* for 5 min at 4°C. Finally, the supernatant was removed, and the cells were resuspended in PBS. The cell suspension was stained with a mixed panel of fluorescent antibodies, including FITC‐conjugated anti‐mouse CD3e (BioLegend, USA, #100305), APC‐conjugated anti‐mouse CD4 (BioLegend, USA, #1001516), and PE‐conjugated anti‐mouse CD8a (BioLegend, USA, #100707), at 4°C for 30 min in the dark. The cells were then centrifuged at 500 × *g* for 5 min at 4°C. After removing the supernatant, the pellet was resuspended in PBS. Flow cytometric data were collected and analyzed using FlowJo v10.8.1, while statistical analysis was conducted with GraphPad Prism 10.1.2.

### 2.10. Immunization Trial of Yaks With BVDV 1u E2 Vaccine Antigen

Fifteen 2–4‐month‐old yak calves were enrolled in the present study at Qinghai Zhonghui Agriculture and Animal Husbandry Technology Co., Ltd. Prior to immunization, all calves were preliminarily screened using the ID Screen BVD p80 Antibody Competition Kit (IDvet Innovative Diagnostics, France, Cat. No. BVDC‐5P) and ID Screen BVD p80 Antigen Capture Kit (IDvet Innovative Diagnostics, France, Cat. No. BVDAGP80‐5P). RT‐PCR targeting the 5ʹ‐UTR of BVDV [22] was also performed to confirm that all calves were negative for BVDV antigen, antibodies, and viral nucleic acid. Recombinant BVDV 1u E2 protein was emulsified thoroughly with the Montanide ISA 201 VG adjuvant (SEPPIC, France) at an equal volume ratio (1:1), yielding a final protein concentration of 125 μg/mL. The 15 qualified yak calves were randomly divided into five groups (*n* = 3 per group) (Table 1). Group 1 was injected with PBS as the negative control. Group 2 received the self‐prepared BVDV 1u E2 subunit vaccine. Group 3 was immunized with a commercial inactivated bivalent vaccine against BVD‐mucosal disease and infectious bovine rhinotracheitis (BVDV‐1 NM01 strain + infectious bovine rhinotracheitis LN01/08 strain, Sinovetah, Jiangsu, China). Group 4 was vaccinated with another commercially available inactivated bivalent vaccine (BVDV‐1 NMG strain + infectious bovine rhinotracheitis LY strain, suspension culture, JINYUBAOLING, Inner Mongolia, China). Group 5 was treated with a commercial inactivated monovalent vaccine against BVD‐mucosal disease (BVDV‐1 NM01 strain, Tecon Biology, Xinjiang, China). All calves were intramuscularly administered with 2 mL of the corresponding vaccine on April 29, May 20, and June 10, 2026, with consistent dosage for three immunizations. Fourteen days after the third vaccination, blood samples were collected from all experimental yaks. The serum was isolated subsequently for the detection and titer determination of serum neutralizing antibodies.

**Table 1:** Immunization grouping in animal trials using yak calves.

Group	Immunogen	Immunization route	Vaccine dosage (mL)	Three‐time immunization schedule		
1	PBS	IM injection	2	April 29	May 20	June 10
2	BVDV 1u E2	IM injection	2	April 29	May 20	June 10
3	BVD‐MD/IBR inactivated bivalent vaccine	IM injection	2	April 29	May 20	June 10
4	BVD‐MD/IBR inactivated bivalent vaccine (suspension culture)	IM injection	2	April 29	May 20	June 10
5	BVD‐MD vaccine	IM injection	2	April 29	May 20	June 10

Abbreviations: BVD‐MD/IBR, bovine viral diarrhea‐mucosal disease and infectious bovine rhinotracheitis; IM injection, intramuscular injection.

### 2.11. Detection of Virus‐Neutralizing (VN) Antibody Titers for BVDV 1u

VN antibody levels were measured using a microtiter virus neutralization test (VNT) in MDBK cells, as described in the WOAH Terrestrial Manual (Chapter 3.4.7) and previously published studies [28, 29]. The challenge strain, BVDV‐2024‐yak‐1 (BVDV 1u genotype, NCP biotype), was isolated from a 1‐year‐old yak with diarrhea and gingival mucosal ulcers in Menyuan County, Haibei Tibetan Autonomous Prefecture, Qinghai Province, China, in August 2024. The isolate caused no obvious CP effect (CPE) in MDBK cells, and it shared the highest nucleotide identity with the Chinese cattle‐derived strain NM2404 (93.01%), followed by the yak‐derived strain M31182 (89.77%) [27]. Briefly, serum samples from the above five groups (group 1–group 5) were first heat‐inactivated at 56°C for 30 min, and serial two‐fold dilutions (1:2–1:1024, 50 µL/well) of individual serum samples were prepared in duplicate wells of 96‐well microtiter plates. Each well received 50 µL of the NCP BVDV 1u yak isolate 2024‐yak1 containing 200 FAID_50_ [27]. After incubation at 37°C for 1 h, 100 µL of an MDBK cell suspension (1 × 10^5^ cells/mL, corresponding to 1 × 10^4^ cells per well) was added to each well, and the plates were incubated at 37°C in a 5% CO_2_ atmosphere for 4–5 days. The cell control consisted of mock‐inoculated MDBK cells incubated with a maintenance medium instead of the virus–serum mixture. The virus control consisted of the virus‐working dilution incubated with 2% horse serum, confirming that the virus‐working dilution was infectious. Infection was detected by indirect immunofluorescence after cell monolayers were fixed and incubated with an anti‐NS3 monoclonal antibody (1:500, clone 4B3, kindly provided by Dr. Wenliang Li, Jiangsu Academy of Agricultural Sciences) and then with Alexa Fluor 488‐conjugated goat anti‐mouse IgG (H + L) (Abcam, 1:1000). Since serum and virus were mixed with equal volumes, the final serum dilution was twice the nominal plate dilution, as specified in the WOAH Terrestrial Manual (Chapter 3.4.7). The VN titers for each serum were therefore expressed as the reciprocal of the highest serum dilution at which the virus was completely neutralized in both duplicate wells (100% neutralization endpoint).

## 3. Results

### 3.1. Bioinformatics Analysis of the E2 Protein

As shown in Table 2, the E2 protein comprises 5,880 atoms, with a molecular formula of C_1892_H_2914_N_492_O_558_S_24_ and a molecular weight of 42.25 kDa, and the protein contains 46 negatively charged residues (Asp + Glu) and 42 positively charged residues (Arg + Lys), with an isoelectric point (pI) of 6.11. The grand average of hydropathicity (GRAVY) was calculated to be −0.300, suggesting that E2 is a hydrophilic protein. Transmembrane region, signal peptide, and hydrophilicity/hydrophobicity analyses demonstrated that the full‐length E2 protein is composed of 374 amino acids. A transmembrane domain was identified at residues 346–368, while no signal peptide was predicted. The protein also possesses alternating hydrophilic and hydrophobic regions. Secondary structure prediction using SOPMA revealed that E2 is dominated by α‐helices, extended strands, and random coils, which account for 13.37%, 28.34%, and 58.29% of the total structure, respectively. Tertiary structure modeling via SWISS‐MODEL indicated a high proportion of random coils, which was consistent with the secondary structure prediction results. Collectively, the bioinformatic analyses revealed that the N‐terminal first 200 amino acids of the E2 protein exhibit favorable hydrophilicity, lack transmembrane regions and signal peptides, and possess favorable folding properties. Multiple B‐cell epitopes are located in this region. Therefore, residues 1–200 aa of E2 were selected as the marker for serological diagnosis. Therefore, the gene fragment encoding amino acids 1–200 of E2 was synthesized by Sangon Biotech (Shanghai) Co., Ltd. The synthesized gene was ligated into the pET‐28a vector to generate the recombinant plasmid pET‐28a‐E2.

**Table 2:** Amino acid composition of the E2 protein.

Amino acids	Number	Frequency (%)	Amino acids	Number	Frequency (%)
Ala (A)	17	4.50	Leu (L)	30	8.00
Arg (R)	15	4.00	Lys (K)	27	7.20
Asn (N)	12	3.20	Met (M)	7	1.90
Asp (D)	24	6.40	Phe (F)	15	4.00
Cys (C)	17	4.50	Pro (P)	18	4.80
Gln (Q)	11	2.90	Ser (S)	9	2.40
Glu (E)	22	5.90	Thr (T)	39	10.40
Gly (G)	31	8.30	Trp (W)	7	1.90
His (H)	8	2.10	Tyr (Y)	20	5.30
Ile (I)	14	3.70	Val (V)	31	8.30

### 3.2. Expression, Purification, and Identification of the Recombinant E2 Protein

Following double restriction enzyme digestion, the recombinant plasmid was separated via 10 g/L agarose gel electrophoresis. A DNA band with the expected size of the E2 gene fragment was detected (Figure 1). Sanger sequencing further verified full consistency with the designed sequence, confirming the successful construction of the recombinant plasmid pET‐28a‐E2. The verified plasmid was transformed into *Transetta* (*DE3*)‐competent cells for protein induction. SDS‐PAGE and western blot analyses showed a specific protein band around 28 kDa (Figure 2A,B). After ultrasonication, the recombinant E2 protein was mainly recovered in the bacterial pellet (Figure 2A), indicating that the recombinant E2 protein was mainly in inclusion bodies. The pellet was denatured with 6 mol/L urea and then subjected to purification, and the target recombinant E2 protein was successfully obtained (Figure 3A). Western blot using BVDV‐positive serum detected a band at the expected molecular weight (Figure 3B), which confirmed that the truncated BVDV 1u E2 protein possessed favorable immunogenicity.

**Figure 1:**
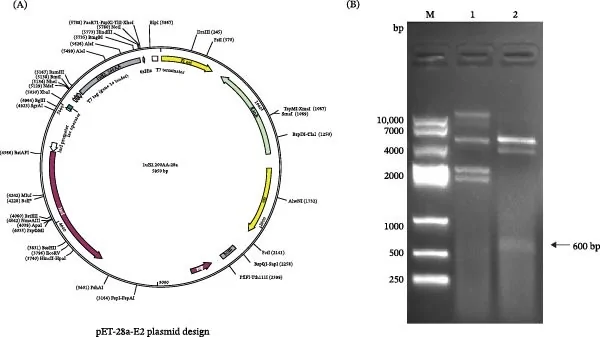
Design and identification of the recombinant plasmid pET‐28a‐truncated E2. (A) Schematic diagram of the recombinant plasmid pET‐28a‐truncated E2. (B) Restriction digestion identification of the plasmid pET‐28a‐truncated E2. M: 10,000 bp DNA marker; 1: undigested recombinant plasmid pET‐28a‐ truncated E2; 2: recombinant plasmid pET‐28a‐truncated E2 after double‐enzyme digestion.

**Figure 2:**
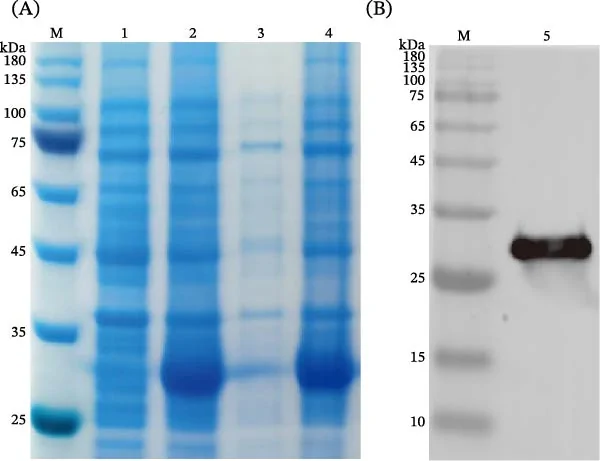
Expression identification of recombinant E2 protein via SDS‐PAGE and western blot. (A) Analysis of expression patterns of the recombinant E2 protein. (B) Reactivity identification of recombinant E2 protein against anti‐His‐tag antibody. M: protein molecular weight marker; 1: whole‐cell lysate of uninduced *E. coli*; 2: whole‐cell lysate of induced *E. coli*; 3: soluble fraction of induced bacterial lysate; 4: inclusion body of induced bacterial lysate; 5: purified recombinant E2 protein.

**Figure 3:**
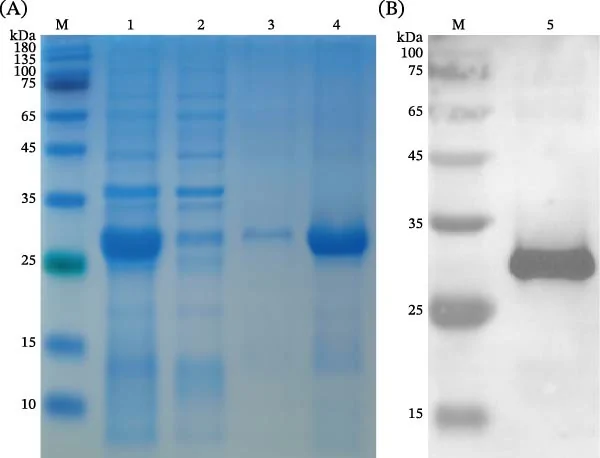
Purification and antigenicity characterization of the recombinant E2 protein. (A) SDS‐PAGE analysis of purified E2 protein. (B) Antigenicity validation of the purified E2 protein with BVDV positive serum by western blot. M: protein molecular weight marker; 1: bacterial supernatant before purification; 2: column flow‐through fluid; 3: eluates with 100 mM imidazole; 4: eluates with 500 mM imidazole; 5: purified recombinant E2 protein.

### 3.3. Development and Clinical Application of an Indirect ELISA for BVDV Antibody Detection

We performed systematic optimization of the indirect ELISA using chessboard titration. The optimized working parameters were determined: antigen coating concentration of 23.60 µg/mL, serum dilution of 1:20 (Table 3), PBST as the blocking reagent (Table 4), and HRP‐conjugated secondary antibody diluted to 1:20000 (Table 5). The incubation steps for serum (Table 6) and HRP‐labeled secondary antibody (Table 7) were both at 37°C for 30 min.

**Table 3:** Determination of the optimal serum dilution.

Serum dilution	Detection results		
	Positive serum OD_450 nm_	Negative serum OD_450 nm_	P/N value
1:25	2.45	0.30	8.07
**1:50**	**2.37**	**0.26**	**9.13**
1:100	1.62	0.24	6.53
1:200	0.53	0.24	2.17

*Note:* Bold values indicate the optimal serum dilution, selected on the basis of the highest P/N ratio.

**Table 4:** Selection of the optimal blocking solution.

Blocking solution	Detection results		
	Positive serum OD_450 nm_	Negative serum OD_450 nm_	P/N value
2% GEL	1.05	0.13	7.95
5% GEL	0.87	0.14	6.09
2% GEL (5% HS)	1.14	0.11	10.53
5% GEL (5% HS)	0.87	0.11	8.00
2% BSA	1.61	0.23	6.90
5% BSA	1.69	0.51	3.28
10% BSA	1.83	0.74	2.47
2% SMP	1.09	0.11	10.13
5% SMP	0.91	0.11	8.72
10% SMP	0.78	0.11	7.49
**PBST**	**1.45**	**0.13**	**10.93**

*Note*: PBST, phosphate‐buffered saline containing 1% Tween‐20.

Abbreviations: BSA, bovine serum albumin; HS, horse serum; SMP, skim‐milk powder.

**Table 5:** Optimal dilution of the HRP‐conjugated secondary antibody.

Dilution of the HRP‐ secondary antibody	Detection results		
	Positive serum OD_450 nm_	Negative serum OD_450 nm_	P/N value
1:5000	3.85	0.63	6.13
1:10,000	2.26	0.29	7.61
**1:20,000**	**1.26**	**0.16**	**7.77**
1:40,000	0.61	0.09	6.37
1:80,000	0.31	0.09	3.18
1:160,000	0.17	0.05	3.11
1:320,000	0.10	0.04	2.18
1:640,000	0.07	0.04	1.70

*Note:* Bold values indicate the optimal dilution of the HRP‐conjugated secondary antibody, selected on the basis of the highest P/N ratio.

**Table 6:** Determination of optimal serum incubation temperature and time.

Serum incubation temperature	Detection results	Incubation time		
		15 min	30 min	45 min
37°C	Positive serum OD_450 nm_	0.81	**1.06**	1.00
	Negative serum OD_450 nm_	0.07	**0.08**	0.08
	P/N value	10.61	**12.11**	11.67

*Note:* Bold values indicate the optimal serum incubation condition, selected on the basis of the highest P/N ratio.

**Table 7:** Determination of the optimal incubation temperature and time for the HRP‐conjugated secondary antibody.

Incubation temperature of HRP‐conjugated secondary antibody	Detection results	Incubation time of HRP‐conjugated secondary antibody		
		15 min	30 min	45 min
37°C	Positive serum OD_450 nm_	2.13	**2.68**	3.04
	Negative serum OD_450 nm_	0.15	**0.17**	0.20
	P/N value	14.22	**15.76**	15.20

*Note:* Bold values indicate the optimal incubation condition for the HRP‐conjugated secondary antibody, selected on the basis of the highest P/N ratio.

ROC analysis based on well‐characterized BVDV‐positive (*n* = 199) and negative (*n* = 264) serum specimens returned an AUC of 0.99 at a PP value of 64.56%, with a standard error of 0.00114 and a 95% CI ranging from 0.98 to 1.00. The Youden index was 0.96, reflecting outstanding performance in distinguishing BVDV‐positive and negative samples. The optimal cutoff value was determined to be 64.56% using MedCalc. At this cutoff, the diagnostic sensitivity was 99.00% (95% CI: 94.60%–98.90%), and the diagnostic specificity was 97.40% (95% CI: 96.40%–99.90%) (Figure 4).

**Figure 4:**
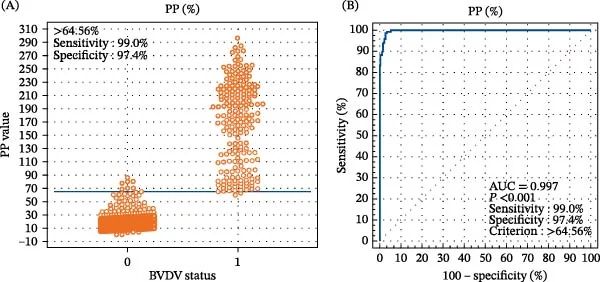
ROC curve analysis of the established ELISA assay. (A) Interactive dot plot for distinguishing BVDV‐positive and BVDV‐negative serum samples from yak (0: BVDV‐negative sera; 1: BVDV‐positive sera). (B) ROC curve displaying the sensitivity and specificity of the ELISA assay.

To evaluate analytical sensitivity, three BVDV‐positive sera were serially diluted. The highest detectable dilution was 1:800 (Figure 5A). The positive serum against bovine *Rotavirus*, infectious bovine rhinotracheitis virus, *P. multocida*, *M. bovis*, and foot‐and‐mouth disease virus was applied for cross‐reactivity testing, and no positive signal was detected (Figure 5B), verifying the high specificity of the assay. Intra‐ and interassay reproducibility was assessed using four serum samples tested in triplicate. All CVs were below 10.00% (Table 8), confirming satisfactory repeatability. Subsequently, 377 field yak serum samples collected from multiple areas in Qinghai Province were screened by the established ELISA. In total, 81 samples were BVDV antibody positive, and 296 were negative, with an overall seroprevalence of 21.48% (Table 9).

**Figure 5:**
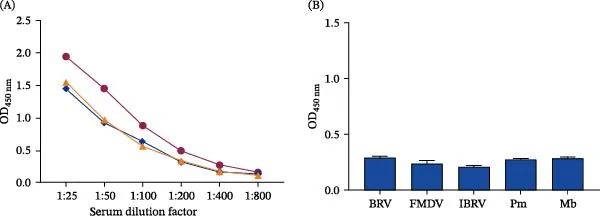
Sensitivity and specificity validation of the indirect ELISA method. (A) Sensitivity analysis using three serially diluted positive yak serum samples. (B) Specificity assessment against sera positive for common bovine pathogens. BRV, bovine *Rotavirus*; FMDV, foot‐and‐mouth disease virus; IBRV, infectious bovine rhinotracheitis virus; Mb, *Mycobacterium bovis;* Pm, *Pasteurella multocida*.

**Table 8:** Repeatability analysis of the indirect ELISA assay based on the E2 protein.

Serum samples	Repeatability assessment	1	2	3	Average	Standard deviation	CV (%)
P1	Intrabatch	3.04	3.05	3.03	3.04	0.01	0.40
P2		3.25	3.22	3.23	3.23	0.01	0.42
N1		0.73	0.75	0.75	0.74	0.01	2.27
N2		0.87	0.86	0.85	0.86	0.00	0.97
P1	Interbatch	3.05	3.08	2.95	3.03	0.06	2.14
P2		3.36	3.27	3.24	3.29	0.06	1.91
N1		0.77	0.71	0.75	0.74	0.03	4.01
N2		0.88	0.86	0.83	0.86	0.02	3.02

**Table 9:** Clinical evaluation of the indirect ELISA method based on the E2 protein.

Province	City (prefecture)	County (district)	Sampling date	Total samples	Positive rate (%)
Qinghai Province	Xining	Datong	2025/4	50	(5/50) 10.00
		Chengzhong	2024/7	18	(17/18) 94.44
		Chengbei	2024/6	5	(4/5) 80.00
		Huangyuan	2025/6	65	(6/65) 9.23
	Haibei	Menyuan	2024/1	18	(5/18) 27.78
		Gangcha	2024/7	5	0
	Yushu	Shang la xiu	2025/5	60	(4/60) 6.67
		Batang	2025/5	3	0
	Goluo	Gande	2024/4	123	(19/123) 15.45
		Maduo	2024/4	20	(11/20) 55.00
		Banma	2024/4	10	(10/10) 100.00
Total	—	—	—	377	(81/377) 21.49

### 3.4. Specificity Identification of Polyclonal Antibodies Against the E2 Protein

Fourteen days following the third immunization, mouse serum samples were isolated and subjected to indirect ELISA for antibody levels. The results revealed that the antibody sensitivity was 1:256,000 for both the E2‐FA and E2‐201 immunization groups (Figure 6). Based on the findings of western blot, the generated E2‐FA and E2‐201 polyclonal antibodies exhibited specific binding to the purified E2 protein, reflecting good specificity (Figure 7A, B). We further assessed antibody reactivity via IFA using BVDV 1u subtype‐infected MDBK cells. Compared with the negative serum control (Figure 7C), green fluorescence was detected in cells incubated with E2‐FA and E2‐201 polyclonal antibodies. Collectively, these data confirmed that both anti‐E2 polyclonal antibodies possessed favorable reactivity with BVDV 1u‐infected cells.

**Figure 6:**
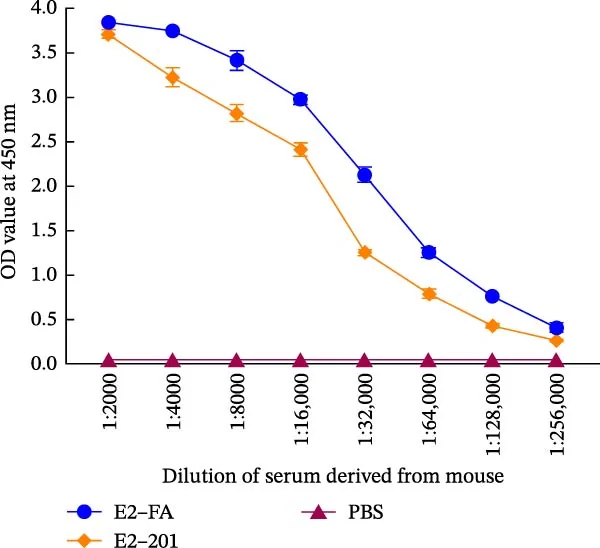
Serum antibody responses in mice immunized with recombinant E2 protein formulated with different adjuvants. Serum antibody levels were determined by indirect ELISA at various serum dilutions. E2‐FA: E2 protein emulsified with Freund’s adjuvant; E2‐201: E2 protein formulated with Montanide ISA 201; PBS: negative control group. OD_450 nm_ values are shown as mean ± standard deviation.

**Figure 7:**
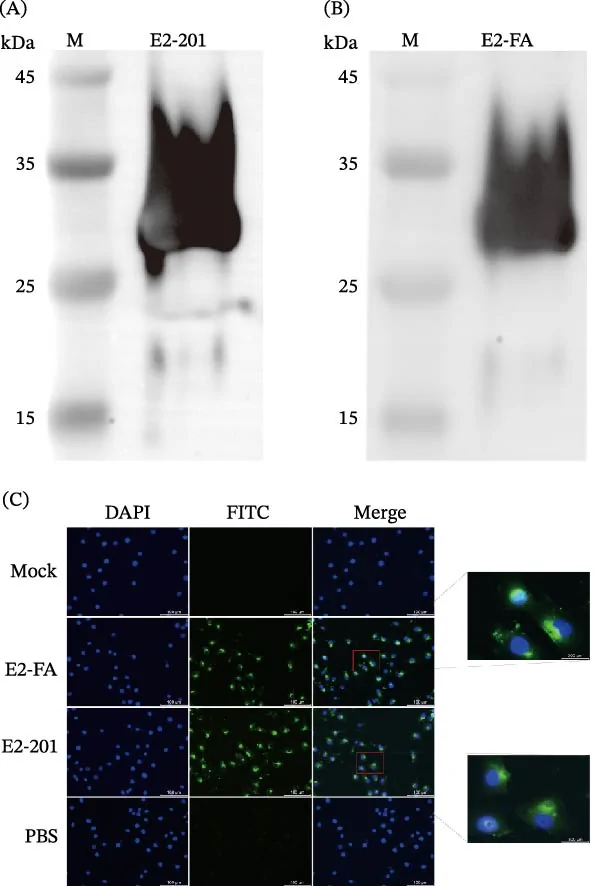
Specificity verification of E2‐derived polyclonal antibodies formulated with different adjuvants. (A, B) Western‐blot identification of specific antibodies derived from E2‐201‐immunized and E2‐FA‐immunized mice with recombinant E2 protein, respectively. M: protein molecular‐weight marker. (C) Immunofluorescence staining for reactivity of BVDV 1u strain to the prepared polyclonal antibody. Mock: mouse negative serum; E2‐FA: polyclonal antibodies from mice immunized with E2 plus Freund’s adjuvant; E2‐201: polyclonal antibodies from mice immunized with E2 plus Montanide ISA 201; PBS: PBS‐immunized negative control. DAPI: nuclear staining; scale bars: 100 μm for main panels; 300 μm for magnified insets.

### 3.5. E2‐201 Promotes Stronger Cellular Immune and Cytokine Responses Than E2‐FA

As shown in Figure 8, the proportions of splenic CD4^+^ and CD8^+^ T lymphocytes were significantly elevated in mice immunized with E2 protein formulated with two adjuvants, indicating that both E2‐201 and E2‐FA successfully triggered T lymphocyte activation. Notably, the E2‐201 group exhibited markedly stronger splenic T‐cell activation than the E2‐FA group (Figure 8). Consistent with the cellular immune responses, detection of the cytokines revealed that the recombinant E2 immunogens both exhibited elevated cytokine production (Figure 9). Furthermore, mice in the E2‐201 group displayed significantly higher cytokine levels compared with those in the E2‐FA group.

**Figure 8:**
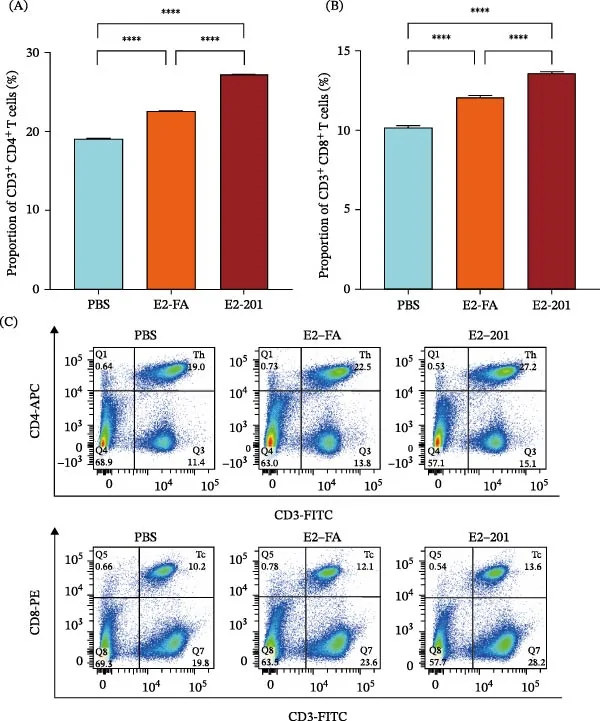
Proportions of CD4^+^ and CD8^+^ T‐cell subsets in splenocytes from immunized mice. (A) Percentage of CD3^+^CD4^+^ T cells. (B) Percentage of CD3^+^CD8^+^ T cells. (C) Representative flow‐cytometry dot‐plots for CD4^+^ and CD8^+^ T‐cell populations. PBS: PBS‐immunized negative control group; E2‐FA: mice immunized with E2 plus Freund’s adjuvant; E2‐201: mice immunized with E2 plus Montanide ISA 201. Data are presented as mean ± standard deviation.  ^∗∗∗∗^, *p* < 0.0001.

**Figure 9:**
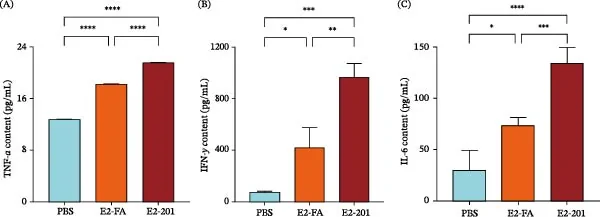
Detection of serum cytokine levels in immunized mice. (A) TNF‐α concentration; (B) IFN‐γ concentration; (C) IL‐6 concentration. PBS: PBS‐immunized negative control group; E2‐FA: mice immunized with E2 plus Freund’s adjuvant; E2‐201: mice immunized with E2 plus Montanide ISA 201. Cytokine levels are expressed in pg/mL. Data are presented as mean ± standard deviation.  ^∗^, *p* < 0.05;  ^∗∗^, *p* < 0.01;  ^∗∗∗^, *p* < 0.001;  ^∗∗∗∗^, *p* < 0.0001.

### 3.6. BVDV 1u‐Specific Neutralizing Antibody Responses Induced by Recombinant E2 Antigen in Yak Calves

As shown in Table 10, serum VN antibody titers against BVDV 1u were detected in yak calves from each immunized group. Calves receiving PBS (group 1) showed no detectable BVDV 1u‐specific neutralizing activity, with all three animals recording titers below 1:4. Animals immunized with recombinant BVDV 1u E2 antigen (group 2) elicited strong neutralizing responses, with ID^#^7132 and ID^#^7104 reaching titers of 1:512, while calf ID^#^7129 had a titer of 1:256. Calves administered the BVD‐MD/IBR inactivated bivalent vaccine (group 3) produced variable antibody levels, with individual titers of 1:128 (ID^#^2048), 1:256 (ID^#^2172), and 1:64 (ID^#^079). For group 4, which received the suspension‐culture‐derived BVD‐MD/IBR inactivated bivalent vaccine, two calves (ID^#^ 7118 and ID^#^ 2103) attained titers of 1:512, whereas one animal (ID^#^2125) had a titer of 1:256. Calves immunized with the BVD‐MD monovalent vaccine (group 5) exhibited titers of 1:128 for ID^#^ 2117 and high titers of 1:512 for the remaining two animals (ID^#^7120 and ID^#^7127). The above findings indicated that recombinant BVDV 1u E2 antigen could induce high‐titer BVDV 1u‐specific neutralizing antibodies in yak calves, with immune responses showing similar trends to those of commercial inactivated vaccines, whereas variable antibody titers were observed among different commercial vaccine groups.

**Table 10:** Detection of virus‐neutralizing antibody titers against BVDV 1u.

Group	Ear‐tag ID number of yak calves	Serum neutralizing antibody titers against BVDV 1u
1‐PBS	7115	<1：4
	2161	<1：4
	7142	<1：4
2‐BVDV 1u E2	7132	1：512
	7129	1：256
	7104	1：512
3‐BVD‐MD/IBR inactivated bivalent vaccine	2048	1：128
	2172	1：256
	079	1：64
4‐BVD‐MD/IBR inactivated bivalent vaccine (suspension culture)	2125	1：256
	7118	1：512
	2103	1：512
5‐BVD‐MD vaccine	2117	1：128
	7120	1：512
	7127	1：512

## 4. Discussion

BVDV has spread globally and caused substantial economic losses to the cattle industry worldwide [30]. Current prevention and control strategies for BVDV can be divided into two main approaches. In low‐prevalence regions, surveillance and eradication programs relying on diagnostic testing and culling are widely implemented. By contrast, integrated management centered on vaccination is adopted in areas with a high BVDV prevalence. Given its global epidemic status, vaccination has become a primary route for BVDV control. Accordingly, developing safe, potent genetically engineered vaccines is critical for the effective prevention and management of BVDV.

As the major structural antigen of BVDV, the E2 protein represents the most potential candidate for the development of diagnostic antigens and subunit vaccines. It contains the dominant and highly conserved antigenic determinants of BVDV and is responsible for mediating viral attachment and entry into host cells. Accordingly, E2‐specific antibodies can efficiently block viral infection and neutralize BVDV infectivity [31]. In comparison, the E0 protein functions as a secreted ribonuclease that suppresses host immune signaling via its RNase activity, enabling the virus to evade interferon‐mediated antiviral responses and thereby interfering with the induction of effective protective immunity [21]. The E1 protein mainly serves an auxiliary function by forming E1‐E2 heterodimers and lacks independent neutralizing epitopes. Numerous previous studies have successfully expressed BVDV structural proteins via prokaryotic and eukaryotic expression systems and further developed serological diagnostic methods and candidate subunit vaccines based on different viral antigens [32–34]. Among multiple BVDV antigen candidates, the E2 protein exhibits satisfactory advantages in sequence conservation, diagnostic specificity, and immune protective efficacy over E0, E1, and other structural proteins, making it the better antigen for BVDV detection and subunit vaccine development [35, 36]. Based on the well‐documented advantages of E2 in terms of antigenicity, biological function, and sequence conservation, the present study adopted a prokaryotic expression system to produce recombinant BVDV E2 protein. Using this purified E2 antigen, we successfully established a reliable indirect ELISA for BVDV antibody detection and further evaluated the immunogenicity of the E2 vaccine antigen by T‐cell activation following mouse immunization and serum neutralizing antibody titers in yak calves, providing important insights into the clinical serological surveillance and subunit vaccine development of BVDV.

In the present study, bioinformatics tools were applied to analyze the physicochemical properties and tertiary structure of the E2 protein from the predominant BVDV 1u subtype circulating in Qinghai yaks. Based on the results of bioinformatics predictions, the truncated fragment covering amino acids 1–200 was selected to construct the prokaryotic expression plasmid (Figure 1). The recombinant E2 protein was successfully expressed in an *E. coli* system and was predominantly produced in the form of inclusion bodies, which is mainly attributable to the intrinsic hydrophobic regions within the E2 protein sequence and the inherent disadvantages of bacterial expression platforms. Unlike eukaryotic systems, *E. coli* cannot perform glycosylation modification to shield hydrophobic domains and lacks a sophisticated molecular chaperone system, thereby hindering correct protein folding and ultimately resulting in inclusion body formation [37]. Although the target E2 protein was initially insoluble, the truncated E2 protein achieved high expression efficiency in the prokaryotic system and possessed good immunogenicity, meeting the requirements for serological diagnostic assay development and immunogenicity evaluation.

The established indirect ELISA assay based on the recombinant E2 protein exhibited favorable comprehensive performance, including a reliable cutoff value, high sensitivity, satisfactory specificity, and stable reproducibility (Tables 2–8; Figure 4). Furthermore, no cross‐reactivity with other common bovine viruses was observed (Figure 5B), confirming the high specificity and practical applicability of the developed assay. Therefore, this ELISA method is suitable for large‐scale serological screening of BVDV infection in yak populations. Clinical sample detection showed that the BVDV antibody positivity rate of yaks in Qinghai Province reached 21.48% (Table 9), which further verifies the high endemic prevalence of BVDV in local yak herds and emphasizes the urgency of strengthening systematic epidemiological surveillance and targeted prevention and control measures in this region.

In this study, the purified recombinant E2 protein was formulated with two different adjuvants and used for mouse immunization to evaluate its immunogenicity. Both the E2‐FA‐ and E2‐201 immunized groups elicited good humoral immune responses, with equivalent high polyclonal antibody levels reaching 1:256,000. In addition, mice from both groups exhibited significantly elevated proportions of splenic CD4^+^ and CD8^+^ T lymphocytes and increased secretion levels of immune‐related cytokines (Figures 8 and 9). These collective results demonstrate that the E2 protein retains favorable immunogenicity and can effectively induce both humoral and cellular immune responses, supporting its potential as a reliable candidate antigen for BVDV subunit vaccine development. Of note, the E2‐201 group generated stronger cellular immune responses and higher cytokine expression than the E2‐FA group, suggesting distinct immune‐modulating differences between the two adjuvants. The relatively limited immune performance of Freund’s adjuvant may be attributed to its inherent defects: its nonmetabolizable oil phase persists in tissues for an extended period and frequently causes local adverse reactions, including redness, swelling, induration, ulceration, and tissue necrosis, thereby triggering excessive and unregulated immune activation [38]. In comparison, adjuvant 201 induces more balanced and moderate immune responses with fewer side effects, superior histocompatibility, and better in vivo metabolizability. Moreover, adjuvant 201 exhibits prominent advantages in maintaining high antibody levels, prolonging immune persistence, improving animal welfare, and ensuring stable experimental reproducibility [39].

More importantly, the recombinant BVDV 1u E2 antigen was capable of eliciting BVDV 1u‐specific neutralizing antibody responses in yak calves (Table 10). Calves immunized with this recombinant immunogen developed better serum neutralizing antibody titers, reaching up to 1:512. These neutralizing responses showed similar trends to those generated by other commercial inactivated vaccines, including the BVD‐MD/IBR bivalent vaccine and the BVD‐MD monovalent vaccine. Notably, considerable inter‐individual variation in neutralizing antibody titers existed within each commercial vaccine group. By contrast, no detectable BVDV 1u‐specific neutralizing activity was found in the PBS‐treated group, with three yak calves showing titers below the detection threshold of 1:4. Therefore, these findings demonstrate that the recombinant E2 antigen can induce potent VN humoral immunity in yak calves, achieving neutralizing antibody titers and showing similar trends to commercially available inactivated vaccines. Nevertheless, the sample size in each immunization group was small (*n* = 3), which limits statistical comparison among different vaccine candidates. Larger‐scale animal trials are therefore required to further evaluate the consistency and magnitude of neutralizing antibody responses induced by this E2 vaccine antigen.

This study has several limitations. First, the truncated E2 antigen may lose partial conformational epitopes, which may impair its immunogenicity. Future study should adopt eukaryotic expression systems, including CHO and HEK293 cells, to generate native‐like E2 protein, further improving the immune‐protective efficacy of the E2‐based subunit vaccine. Second, although serum neutralization assays were performed in the present study, viral challenge experiments and evaluation of long‐term immune persistence were not performed. Therefore, subsequent work should complement challenge trials with BVDV 1u and other relevant strains to confirm the protective potency of the E2 vaccine antigen. Finally, neutralizing antibody titers were only determined against BVDV 1u; serum neutralization assay was not detected for other strains such as BVDV‐1a, BVDV‐2, and BVDV‐3. Sera obtained in this study can be utilized in subsequent work to assess neutralizing activity against these additional strains, helping to determine whether the BVDV 1u E2 immunogen can elicit cross‐protective immunity.

## 5. Conclusion

In the present study, the N‐terminal truncated E2 fragment (amino acids 1–200) of Qinghai yak‐prevalent BVDV 1u was successfully expressed and retained favorable immunogenicity and established an indirect ELISA for BVDV antibody detection featuring high sensitivity‐specificity, acceptable repeatability, and negligible cross‐reactivity. Mouse immunization verified that recombinant E2 elicited both humoral and cellular immunity; compared with Freund’s adjuvant, Montanide ISA 201 can induce stronger T‐cell and proinflammatory cytokine responses. In BVDV‐naïve yak calves, E2‐ISA 201 induced BVDV 1u‐specific neutralizing antibody titers, showing similar trends to commercial inactivated vaccines. Further large‐scale animal work, viral challenge trials, and cross‐genotype neutralization assays are needed to define its protective capacity and cross‐reactive potential, offering experimental evidence for targeted BVDV epidemic prevention and control in yak farming regions.

## Author Contributions

Zhi Li and Yong Fu conceived the study and reviewed and edited the manuscript. Ru Meng, Zhi Li, and Shandian Gao designed the methodology. Jie Li, Ru Meng, Yuan Han, Jinshu Sui, Shuai Wang, and Zhi Li performed the investigation, visualization, and supervision. Jie Li, Zhi Li, and Yong Fu wrote the original draft.

## Funding

The research was supported by grants from the Key R&D and Transformation Plan of Qinghai Province (Grant 2024‐NK‐103).

## Disclosure

All authors read and approved the final article and provided the corresponding authors with written permission to be named in the article.

## Ethics Statement

The animal study was approved by the Institutional Animal Care and Use Committee of the Qinghai University (PJ202401‐69). The study was conducted in accordance with local legislation and institutional requirements.

## Conflicts of Interest

The authors declare no conflicts of interest.

## Data Availability

The data that support the findings of this study are available from the corresponding authors upon reasonable request. The data that support the findings of this study are not publicly available due to restrictions concerning the source of the animal sera.
